# Time-encoded bio-fluorochromic supramolecular co-assembly for rewritable security printing†

**DOI:** 10.1039/d1sc03105h

**Published:** 2021-07-01

**Authors:** Zhao Gao, Shuai Qiu, Fei Yan, Shuyi Zhang, Feng Wang, Wei Tian

**Affiliations:** Shaanxi Key Laboratory of Macromolecular Science and Technology, MOE Key Laboratory of Material Physics and Chemistry Under Extraordinary Conditions, School of Chemistry and Chemical Engineering, Northwestern Polytechnical University Xi'an 710072 P. R. China happytw_3000@nwpu.edu.cn; CAS Key Laboratory of Soft Matter Chemistry, iChEM (Collaborative Innovation Center of Chemistry for Energy Materials), Department of Polymer Science and Engineering, University of Science and Technology of China Hefei Anhui 230026 P. R. China

## Abstract

Innovative fluorescence security technologies for paper-based information are still highly pursued nowadays because data leakage and indelibility have become serious economic and social problems. Herein, we report a novel transient bio-fluorochromic supramolecular co-assembly mediated by a hydrolytic enzyme (ALP: alkaline phosphatase) towards rewritable security printing. A co-assembly based on the designed tetrabranched cationic diethynylanthracene monomer tends to be formed by adding adenosine triphosphate (ATP) as the biofuel. The resulting co-assembly possesses a time-encoded bio-fluorochromic feature, upon successively hydrolyzing ATP with ALP and re-adding new batches of ATP. On this basis, the dynamic fluorescent properties of this time-encoded co-assembly system have been successfully enabled in rewritable security patterns *via* an inkjet printing technique, providing fascinating potential for fluorescence security materials with a biomimetic mode.

## Abstract 2

Rewritable security printing has been successfully achieved based on a biofuel-driven transient supramolecular co-assembly mediated by an enzyme, providing fascinating potential for artificial functional materials with a biomimetic mode.

## Graphical abstract

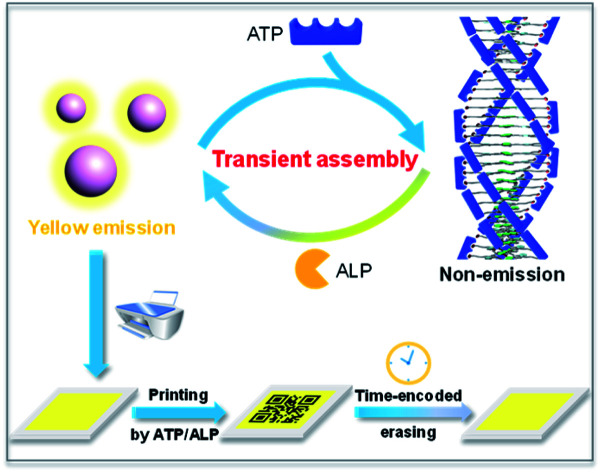

## Introduction

Paper document security is of paramount importance even in the electronic information world, because the widespread medium for documentation is still paper.^1–7^ Developing innovative confidentiality methods for genuine documents is in great demand.^8–10^ In this respect, researchers are interested in utilizing fluorescent security inks to achieve this goal, because of their visibility only under UV light.^11–15^ Nevertheless, most of the reported fluorescent inks are poor in complexity and tunability, which are relatively easy to replicate. Additionally, the printed information is usually not erasable, and the paper can only be used once. Hence, it is highly desirable to develop advanced fluorescent materials with security printing and rewritable features.

To attain these objectives, a feasible strategy is to exploit various fluorophores with stimuli-responsiveness by chemical synthesis.^16–19^ However, the disadvantage of this strategy is that rare responsive units are available. An ingenious approach is to dynamically regulate the packing mode of π-conjugated molecules in a noncovalent self-assembly way, resulting in a reversible change of fluorescence intensity and wavelength.^20–23^ In this respect, fluorochromic supramolecular assemblies triggered by heat, light, solvent, vapor and force have drawn great enthusiasm.^24–28^ Among them, bio-fluorochromic assembly is of particular interest, because of its possible transient characteristics in response to biological sources, which can be regarded as a superior candidate for smart fluorescent materials. Nevertheless, reports highlighting bio-fluorochromic assembly and biofueled transient assembly^29–34^ in one system have been rarely exploited so far, but need to be addressed because they are closer to the naturally occurring system and more efficient in some specific scenarios. Here, we propose a simple co-assembly strategy to build a novel transient bio-fluorochromic system, where the emission signals of π-conjugated units can be reversibly modulated on a time scale, nicely meeting the color variability and repeatability requirements for exploiting in rewritable security printing.

**Fig. 1:**
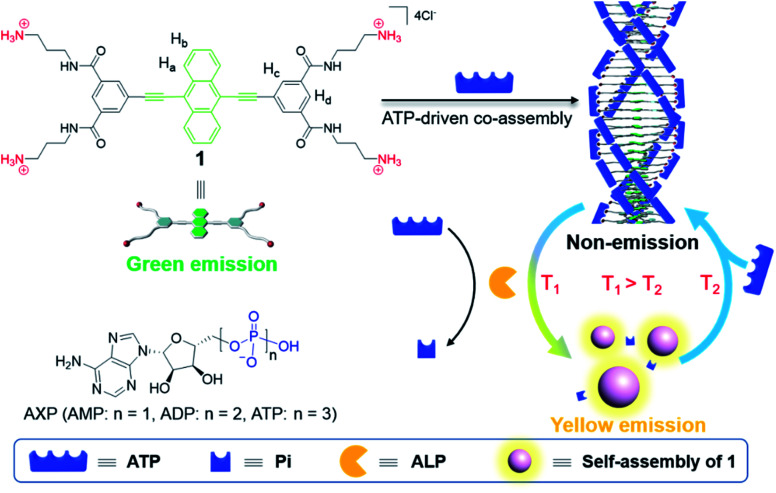
Schematic illustration of tetrabranched cationic diethynylanthracene monomer **1** and AXP, and cartoon representation for the ATP-driven transient co-assembly mediated by ALP. (AMP: adenosine monophosphate, ADP: adenosine diphosphate, ATP: adenosine triphosphate, ALP: alkaline phosphatase, and Pi: monophosphate).

As a proof of concept, we present a transient bio-fluorochromic supramolecular co-assembly that can be mediated by an enzyme with a tunable emission signal. Specifically, a tetrabranched cationic diethynylanthracene monomer **1** has been firstly designed and synthesized (Fig. 1). It can self-assemble into nano-micelles in aqueous medium, accompanied by dramatically red-shifted fluorescence. Upon adding a biofuel of adenosine triphosphate (ATP) into **1**, supramolecular co-assembly **1**/ATP tends to form, accompanied by the quenching of the emission signal. Gratifyingly, the co-assembly **1**/ATP can be transiently destroyed by temporally regulating the consumption of ATP using a hydrolytic enzyme (ALP: alkaline phosphatase), which is similar to the naturally occurring self-assembled cytoskeleton proteins in living cells.^35,36^ Thus, time-encoded fluorescence is generated in the synthetic assembled system, *via* repeatedly adding new batches of ATP (Fig. 1). On this basis, the resulting transient bio-fluorochromic supramolecular assembly can be used as a security ink to develop rewritable security printing materials with a unique time-encoded feature, which highly improves the security and repeatability of paper-based confidential information.

## Results and discussion

### Monomer design

The structure of desired monomer **1** is shown in Fig. 1, which contains two sets of symmetrical propyl ammonium salt groups that are linked to the periphery of the 9,10-bis(phenylethynyl)anthracene unit *via* amide bonds. The branched ammonium cation is in charge of solubility in aqueous solution. More importantly, it favors the formation of electrostatic interaction and hydrogen bonding between the R–NH_3_^+^ of positively charged **1** and R–PO_3_^2−^ of negatively charged ATP, resulting in the formation of supramolecular co-assembly with chirality transfer from ATP to **1**. 9,10-Bis(phenylethynyl)anthracene, the core of **1**, possesses red-shifted fluorescence in the aggregated states, which is suitable for being employed to construct fluorochromic materials.^37,38^ Detailed synthetic procedures and characterization results are shown in the ESI.†

### Self-assembly behaviors of **1**

**Fig. 2:**
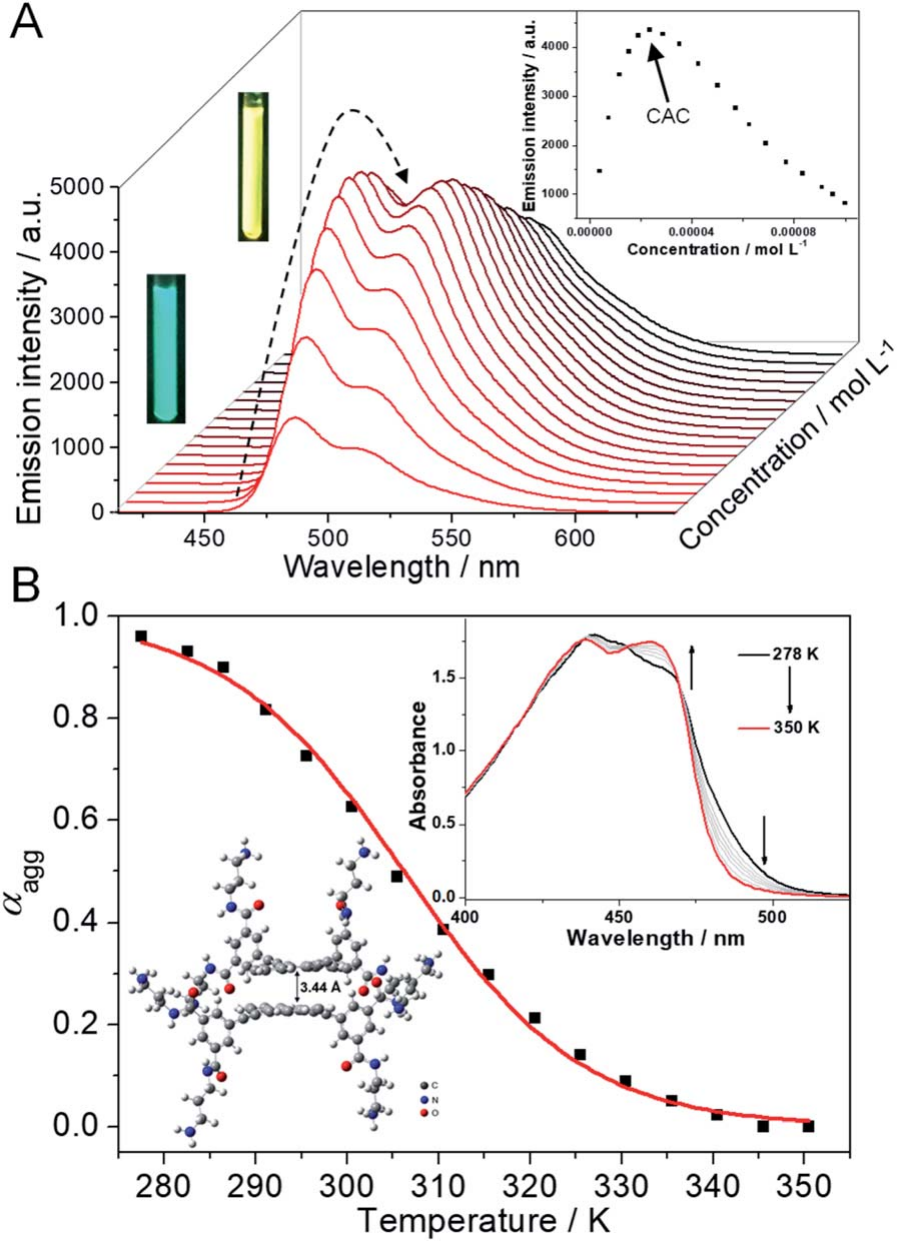
Self-assembly behaviors and mechanism of **1**. (A) Concentration-dependent fluorescence spectra of **1** in aqueous solution from 4.0 × 10^−6^ M to 1.0 × 10^−4^ M. *λ*_ex_ = 320 nm. Inset of (a): the plot of the emission intensity of **1** at 484 nm *versus* concentration. (B) *α*_agg_ of **1** (1.2 × 10^−4^ M, *λ* = 490 nm) *versus* temperature in aqueous solution. The red line denotes the isodesmic fitting curve. Inset of (B): temperature-dependent UV-Vis spectra of **1**. Arrows indicate the spectral variations upon increasing temperature. Optimized geometries of the dimeric structures of **1** in the front view, on the basis of DFT calculations at the level of B3LYP/6-31G(d).

Initially, the supramolecular self-assembly behavior of **1** is carefully investigated. In dilute aqueous solution, the UV-Vis spectra of **1** show two major absorption bands in the regions of 300–325 nm and 350–500 nm with vibronic fine structures, indicative of molecularly dissolved states (ESI Fig. S1A,† black line).^39,40^ Upon gradually increasing the concentration of **1**, the molar extinction coefficient (*ε*) at around 460 nm gradually decreases, while a red-shifted shoulder band located between 470 and 530 nm appears (ESI Fig. S1A†). Two distinctive isosbestic points at 471 and 375 nm suggest the transition from a molecularly dissolved state to a self-assembled state. The concentration-dependent fluorescence spectra of **1** are then studied. An emission band centered at 484 nm, along with a vibronic peak at 513 nm, is observed at a diluted concentration (green emission color, Fig. 2A), which is a typical emission attributed to the monomeric 9,10-bis(phenylethynyl)anthracene units. Upon increasing the concentration of **1**, the emission band at 484 nm increases firstly, and then decreases to disappear, and a structureless emission band located at 517 nm gradually turns into the main band (Fig. 2A, and ESI Fig. S1B†). The emission color varies from green to yellow upon increasing the concentration (Fig. 2A, inset), indicative of a self-assembled state of **1**. The plot of the emission intensity at 484 nm *versus* concentration reveals that the critical aggregation concentration (CAC) is 2.4 × 10^−5^ M (Fig. 2A, inset). Dynamic light scattering (DLS) and transmission electron microscopy (TEM) measurements are then employed, which reveal that nano-aggregates (∼60 nm DLS hydrodynamic diameter) are obtained for **1** in aqueous solution (ESI Fig. S2†).

The self-assembly mechanism of **1** is further elucidated *via* temperature-dependent UV-Vis spectral measurements. In detail, the absorption spectra of **1** (1.2 × 10^−4^ M) display two isosbestic points (452 and 469 nm) upon heating (Fig. 2B, inset), suggesting the transition between self-assembled and monomeric states. When monitoring the fraction of aggregated species (*α*_agg_, *λ* = 490 nm) *versus* temperature, a sigmoidal melting curve is obtained (Fig. 2B, and ESI Fig. S3†), which demonstrates the involvement of an isodesmic mechanism in the self-assembly process of **1**.^41–43^ Non-linear fitting of the melting curve affords a Δ*H* (enthalpy release upon aggregation) value of −87.5 kJ mol^−1^, together with a *T*_m_ (melting temperature at which *α*_agg_ is 0.5) value of 306.2 K (Table S1†). According to the modified van't Hoff plot, the Δ*G* (Gibbs free energy) value of the self-assembly process of **1** is calculated to be −24.9 kJ mol^−1^ at 298 K (ESI Fig. S4†). Thus, combining with density functional theory (DFT) calculations at the level of B3LYP/6-31G(d) (Fig. 2B, inset), we rationalized that, driven by the intermolecular π–π stacking and hydrophobic interactions,^44–46^**1** self-assembles into small nano-aggregates in aqueous solution *via* an isodesmic mechanism.

### ATP-driven supramolecular co-assembly

**Fig. 3:**
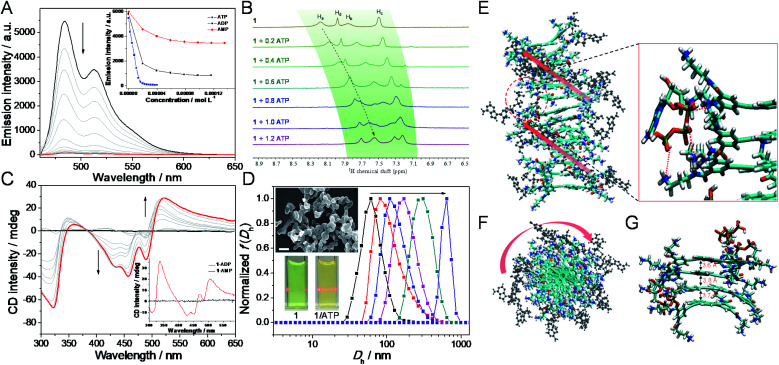
Biofuel-driven supramolecular co-assembly. (A) Fluorescence, (B) partial ^1^H NMR (400 MHz, 298 K, and 2.0 mM of **1** in D_2_O in the presence of 0–1.2 equiv. of ATP), (C) CD spectra and (D) DLS curve variations upon gradual addition of ATP (0–2.0 × 10^−5^ M) into the aqueous HEPES (2-[4-(2-hydroxyethyl)piperazin-1-yl]ethanesulfonic acid) buffer of **1** (2.0 × 10^−5^ M). Inset of (A): the emission intensity changes at *λ* = 484 nm with the addition of ATP, ADP and AMP into **1**, respectively. *λ*_ex_ = 420 nm. Inset of (C): CD spectra of **1**/ADP ([**1**] : [ADP] = 1 : 2) and **1**/AMP ([**1**] : [AMP] = 1 : 4). Inset of (D): photographs of **1** and **1**/ATP ([**1**] : [ATP] = 1 : 1) showing a Tyndall effect. SEM micrograph of **1**/ATP ([**1**] : [ATP] = 1 : 1). Scale bar: 500 nm. (E) Side view and (F) top view of the structure of co-assembly **1**/ATP obtained from MD simulations in the final snapshots. **1** moieties are depicted in colored thick sticks and ATP in black ball-sticks. The arrows indicate the direction of the helix. Zoomed image in (E) shows intermolecular hydrogen bonding between **1** and ATP as red dashed lines. (G) MD simulation of the **1**/ATP tetramer showing intermolecular π–π interactions.

After elucidating the self-assembly behaviors of **1** (1.2 × 10^−4^ M), we then sought to investigate the co-assembly of **1** with ATP. Intriguingly, as ATP is progressively introduced into the solution of **1** (2.0 × 10^−5^ M),^47^ the original absorption bands located between 350 and 550 nm gradually decrease, while a new set of bands centered at 457 nm emerges with an isosbestic point at 479 nm (ESI Fig. S5†). Simultaneously, the addition of ATP to **1** reduces the fluorescence intensity until complete quenching (Fig. 3A), which is a result of large aggregate formation of **1**/ATP (*vide infra*).^48^ Detailed titration experiments reveal that around one equivalent of ATP is required to bind with **1** (Fig. 3A, inset, and ESI Fig. S5†). These phenomena evidence that ATP is able to induce the formation of co-assembly of **1**/ATP in aqueous media.

To get further insights into the co-assembly process of **1**/ATP, various experimental techniques are then employed. Upon mixing **1** and ATP equivalently in D_2_O (2.00 mM for each component), the resonances on **1** and ATP shift up-field (Δ*δ* = 0.57, 0.52, 0.25, and 0.26 for H_a–d_ on **1**, and 0.91 ppm for H_3_ on ATP, respectively), while the well-defined signals become broad (ESI Fig. S6A†). ^1^H NMR titration measurements show that the aromatic protons on **1** gradually up-field shift after doping with ATP (Fig. 3B, and ESI Fig. S6B and C†). Only one set of aromatic signals for **1** is present, revealing the involvement of fast-exchanging noncovalent interactions between **1** and ATP on the NMR time scale.^49^ Circular dichroism (CD) is then considered to reveal the gradual evolution of Cotton effects of **1**/ATP. The CD signals progressively reach a plateau with an isodichroic point at 383 nm (for 457 nm, Δ*ε* = −59.6 L mol^−1^ cm^−1^ and anisotropy factor *g* = −0.0062; for 489 nm, Δ*ε* = −37.6 L mol^−1^ cm^−1^ and *g* = −0.0042; for 521 nm, Δ*ε* = 43.0 L mol^−1^ cm^−1^ and *g* = 0.0122, Fig. 3C and ESI Fig. S7A†), which suggests that ATP induces a preferred supramolecular chirality to the resulting co-assembly of **1**/ATP. A negligible linear dichroism (LD) signal can be detected for **1**/ATP (ESI Fig. S7B†), indicating that the measured CD signals are real to reflect the supramolecular chirality, without the interference of LD.^50^ Additionally, DLS spectra titration reveals that the size gradually increases when continually adding ATP into **1**, accompanied by the emergence of the Tyndall effect (Fig. 3D, and inset). TEM and scanning electron microscopy (SEM) images show that the morphologies of **1**/ATP are nanostructures with a width of 40 nm and mutually entangled to form large-sized aggregates (Fig. 3D, inset, and ESI Fig. S8†), which is in stark contrast to the morphological size of the self-assembly of **1**.

For the purpose of obtaining deeper mechanistic insight into the ATP-driven co-assembly with **1**, molecular dynamics (MD) simulations are performed. The simulation box of the co-assembly **1**/ATP containing 14 monomers **1** and 14 ATP molecules in explicit water solvent is calculated based on a CHARMM36 force field at 298 K for 20 ns to equilibrate the initial configuration (ESI Fig. S9†). As shown in the side and top views of **1**/ATP in the final snapshots (Fig. 3E and F), the supramolecular *M*-helical structure is composed of the 9,10-bis(phenylethynyl)anthracene planar cores and the chiral adenosine lying at the periphery of the aggregates, through the intermolecular electrostatic and hydrogen-bonding interactions between the R–NH_3_^+^ units in **1** and phosphates in ATP. All three of the phosphates in ATP participate in the binding with **1** (the zoomed image in Fig. 3E, shown as red dashed lines). We thus rationalized that the binding of one equivalent of ATP causes the core of **1** to stack with each other by π–π interactions (average value of π–π distances is found to be 3.7 Å, Fig. 3G), which synergistically stabilizes the co-assemblies **1**/ATP and endows them with well-defined structures.

Compared to the co-assemblies of **1**/ATP induced by ATP, the addition of similar diphosphate (ADP: adenosine diphosphate) or monophosphate (AMP: adenosine monophosphate) leads to a different co-assembly behavior from that of **1**/ATP. Specifically, around two equivalents of ADP are required to totally change the emission and absorbance signals (Fig. 3A, inset, and ESI Fig. S10A and B†). Although ADP can also induce CD signals with an isodichroic point at 397 nm, they are less intense than that of ATP (Fig. 3C, inset, and ESI Fig. S10C†). As both of the negative and positive maxima of CD signals are located in the same region for **1**/ADP and **1**/ATP, respectively, **1**/ADP should possess the same helical handedness with **1**/ATP. Only small aggregations are formed for the resulting co-assembly **1**/ADP, as evidenced by the TEM and DLS measurements (ESI Fig. S10D†). Unfortunately, even though an excess of AMP is added into **1**, insignificant CD and emission spectral changes are observed (ESI Fig. S11†). Considering that the monophosphate AMP cannot induce ordered supramolecular structures,^33,51^ it is thus envisioned that the multivalent and chelation effect of non-covalent interactions between the positively charged ammonium groups of **1** and the negatively charged phosphates of ATP play a crucial role in inducing structurally well-defined supramolecular co-assembly **1**/ATP.

### Transient bio-fluorochromic assembly

**Fig. 4:**
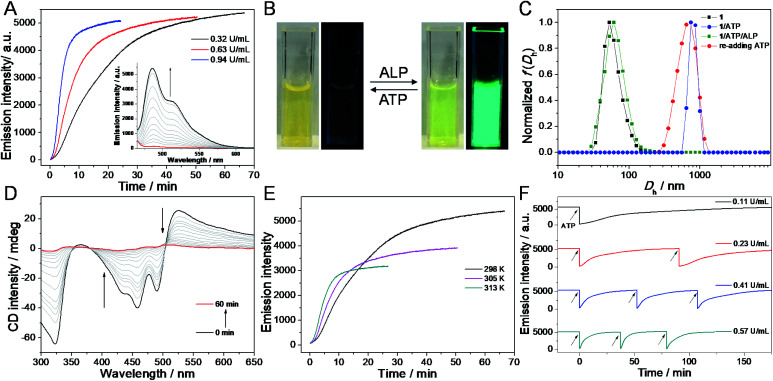
ALP mediated transient co-assembly of **1**/ATP. (A) Fluorescence intensity of **1**/ATP at 484 nm *versus* hydrolysed time with different concentrations of ALP at 298 K. Inset of (A): fluorescence spectral variations of **1**/ATP *versus* hydrolysed time in the presence of 0.32 U mL^−1^ of ALP. *λ*_ex_ = 420 nm. (B) Reversible emission color transition of **1** upon successive addition of ATP and ALP. (C) DLS data showing the induced assembly of **1** by ATP and disassembly by 0.32 U mL^−1^ of ALP. (D) CD spectral variations of **1**/ATP *versus* hydrolysed time in the presence of 0.32 U mL^−1^ of ALP. (E) Fluorescence intensity of **1**/ATP at 484 nm *versus* hydrolysed time with 0.32 U mL^−1^ of ALP at varying temperature. (F) Time-dependent fluorescence intensity at 484 nm upon several repetitive additions of ATP to **1** in the presence of 0.11 U mL^−1^, 0.23 U mL^−1^, 0.41 U mL^−1^ and 0.57 U mL^−1^ of ALP, respectively. Arrows represent the (re)fueling points with the addition of ATP. ([**1**] = 2.0 × 10^−5^ M, [ATP] = 2.0 × 10^−5^ M, aqueous HEPES buffer, and pH = 7.0).

Before investigating the transient bio-fluorochromic co-assembly of **1**/ATP mediated by an enzyme, we firstly explore the enzyme induced disassembly capacity upon *in situ* decomposition of ATP. ALP, a kind of enzyme obtained from bovine intestinal mucosa, is chosen as the enzymatic cleavage reagent, because it can hydrolyse ATP to adenosine and three equivalents of monophosphates (Pi) under mild conditions.^52^ Inasmuch as the hydrolysates cannot induce **1** to assemble into co-assemblies, **1**/ATP would disassemble along with the hydrolysis of ATP. When ALP is added to the co-assembly of **1**/ATP, the intensity of fluorescence spectra gradually increases up to the initial intensity of that of monomer **1**, indicative of the ALP induced disassembly and release of free monomers (Fig. 4A). Simultaneously, the emission color of the solution mixture recovers to the original green (Fig. 4B). Further evidence for ALP induced disassembly is observed by DLS measurements, showing a significant decrease in the aggregate size (Fig. 4C). Time-dependent CD spectra show a weakening trend during the enzymatic process of **1**/ATP by ALP (Fig. 4D, ESI Fig. S12A and B†), which clearly indicates the gradual disassembly of the chiral aggregates.

Furthermore, the kinetics of the disassembly process of **1**/ATP with varying enzyme units presents accelerated emission recovery with increasing concentration of ALP. When the amount of ALP increases from 0.32 U mL^−1^ to 0.63 U mL^−1^ and 0.94 U mL^−1^, the recovery half-life time (defined as the time required to reach 50% of the fluorescence intensity) shortens from 14.5 min *via* 7.3 min to 3.6 min (Fig. 4A, and ESI Fig. S12C†). The corresponding rate constants of disassembly are calculated to be 0.06 min^−1^, 0.12 min^−1^, and 0.29 min^−1^. The results unambiguously prove that the rate of the enzymatic hydrolysis reaction for ATP directly reflects the progress of the disassembly process. Additionally, the rate of the disassembly process of **1**/ATP could also be regulated by the temperature of enzymatic hydrolysis, which demonstrates a faster disassembly process at a higher temperature (Fig. 4E).

With the re-addition of ATP into the solution mixture, the green emission color is immediately quenched, accompanied by fluorescence spectra disappearance (Fig. 4B). Meanwhile, the DLS hydrodynamic diameter and Cotton effects are totally restored to the initial state (Fig. 4C, and ESI Fig. S12D†). Such phenomena manifest that the co-assembly **1**/ATP is re-formed. More interestingly, the fluorescent signal reappears spontaneously with time increasing, which is ascribed to the presence of ALP in the solution system. Subsequently, with the addition of ATP again, the same trend as the previous cycle is observed. With continuously adding new batches of ATP, the emission signal can be reversibly switched for multiple cycles (Fig. 4F). Furthermore, the transient bio-fluorochromic cycles of co-assembly **1**/ATP are confirmed at a different order of ALP units. Upon increasing the amount of prestored ALP in **1**/ATP, the shortened half-life times of the transient cycles are observed from the time-dependent fluorescence spectra (Fig. 4F). The same trend is also obtained from the absorption signals (ESI Fig. S13†). Hence, a transient bio-fluorochromic co-assembly mediated by an enzyme is successfully established, with time-encoded dynamic fluorescent properties.

### Rewritable security printing

**Fig. 5:**
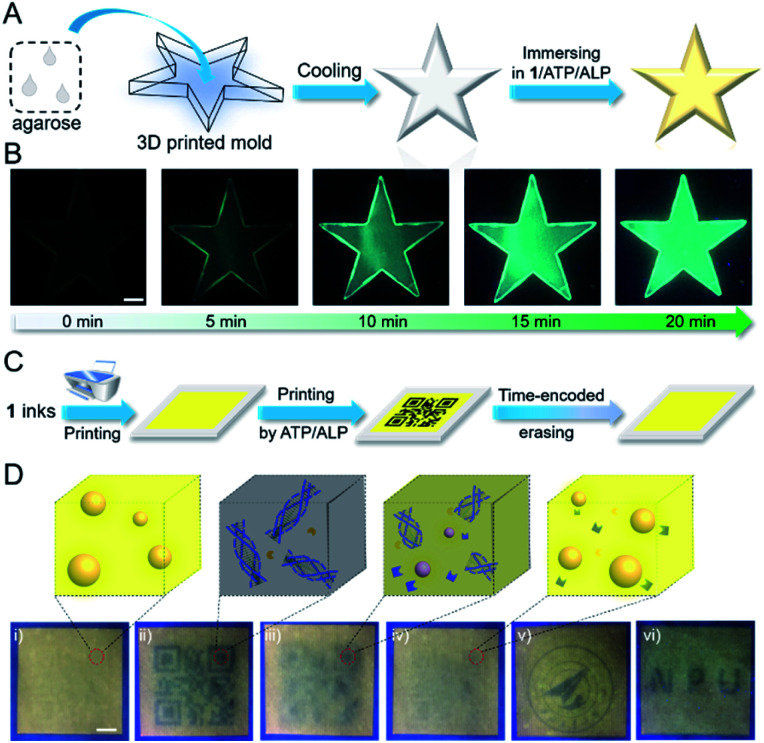
Rewritable security printing from **1**/ATP/ALP. (A) Cartoon representation of the fabrication of the star-shaped 3D model. (B) Photographs of the prepared 3D model with time-dependent emission enhancement behavior (0–20 min), derived from the transient co-assembly of **1**/ATP mediated by ALP ([**1**] = [ATP] = 2.0 × 10^−5^ M and [ALP] = 0.80 U mL^−1^). Scale bar: 5 mm. (C) Schematic illustration of the rewritable security printing. (D) Photographs of the printed patterns: (i) the pattern printed with the aqueous solution of **1** as the ink; (ii) the QR code pattern printed with the inks of ATP/ALP at the same location of (i); (iii) and (iv) the time-encoded erasing of the patterns for 5 min and 10 min, respectively; (v) the reprinted logo pattern with the inks of ATP/ALP at the same location of (iv); (vi) the reprinted “NPU” pattern with ATP/ALP inks ([**1**] = [ATP] = 2.0 × 10^−5^ M, 0.80 U mL^−1^). Scale bar: 5 mm.

Considering that the emission signals of **1**/ATP can be fine-tuned with ALP, it can be employed for developing time-encoded bio-fluorochromic materials. To verify this assumption, a 3D model of fluorescence changing with time is fabricated firstly. In detail, 2 wt% agarose aqueous solution is heated to clarify, and then transferred into a 3D printed mold (Fig. 5A). A non-fluorescent star-shaped model is obtained after immersing it into the mixed solution of **1**/ATP/ALP for several minutes. When the model is standing at room temperature, the green emission color gradually emerges within 20 min (Fig. 5B). This spontaneous emission variation of the hydrogel system on a time scale is highly plausible, since **1**/ATP disassembles in the hydrogel along with the enzyme-catalyzed hydrolysis process of ATP.^53^ Thus, it is evident that the ALP mediated transient assembly of **1**/ATP can be regarded as an effective strategy for the construction of time-encoded emission systems.

On this basis, we then turn to explore its potential application in rewritable security printing. Specifically, the aqueous solution of **1** is firstly filled into the customized cartridge of an inkjet printer (Fig. 5C and D). A square yellow emission area is printed on a non-fluorescent paper. Then, a QR code pattern can be printed in this yellow emission area, by loading with the mixture of ATP/ALP aqueous solution as an ink. The phenomenon is attributed to the co-assembling of **1** and ATP, resulting in the fluorescence quenching of the printed location on the paper. The QR code is invisible under natural light, but can be decoded under UV light by using a smartphone. Interestingly, the temporary QR code is gradually erased with time increasing, which fails to be recognized after 10 min. Such results are ascribed to the presence of ALP in the printed area, which destroyed the co-assembly **1**/ATP by hydrolysing ATP. Thus, the ATP/ALP aqueous solution can be used as a transient ink to transfer messages with time-encoded features in security papers. Furthermore, upon reprinting with ATP/ALP inks, the writing/erasing process can be performed multiple times (Fig. 5D). Moreover, the erasing time of the rewritable information can be regulated according to the concentration of ALP inks, endowing with anti-counterfeiting ability in the time dimension (ESI Fig. S14 and S15†). The rewritable security paper possesses enough stability under ambient conditions, as evidenced by the unchanged emission color for at least half a year (ESI Fig. S16†). Overall, the time-encoded bio-fluorochromic co-assembly **1**/ATP possesses temporal emission signals mediated by ALP on a time scale, which is suitable for multiple-time uses in security printing.

## Conclusions

In summary, we have successfully established a transient bio-fluorochromic supramolecular system through the electrostatic and hydrogen bonding interactions between the tetrabranched cationic diethynylanthracene monomer **1** and anionic biofuel ATP. The nonequilibrium state of co-assembly **1**/ATP can be finely controlled in aqueous medium by temporally regulating the consumption of ATP using its hydrolytic enzyme ALP, accompanied by fluorescence signal variations. Furthermore, the resulting bio-fluorochromic system is exploited to achieve rewritable security printing, by taking advantage of its time-encoded multiple-cycle emission switching features. Hence, the present work not only opens up a new avenue towards artificial functional materials with a biomimetic mode, but also greatly improves the reusability and security level of paper-based confidential information.

## Data availability

Detailed synthetic procedures, analytical data, and computational methods are provided in the ESI.

## Author contributions

Z. G. and W. T. conceived the idea for this project. S. Q., F. Y. and S. Z. performed the experiments, analyzed the data, and produced the artwork under the direction of Z. G. and W. T. All authors contributed to the manuscript preparation.

## Conflicts of interest

There are no conflicts to declare.

## Supplementary Material

SC-012-D1SC03105H-s001
