# Supplementary material for: Time-encoded bio-fluorochromic supramolecular co-assembly for rewritable security printing
Source: Chem Sci. 2021 Jul 1;12(29):10041–7. doi: 10.1039/d1sc03105h (PMC8317669; doi:10.1039/d1sc03105h)
Supplement: SC-012-D1SC03105H-s001 [file SC-012-D1SC03105H-s001.pdf]

Electronic Supplementary Material (ESI) for Chemical Science.
This journal is © The Royal Society of Chemistry 2021

Electronic Supplementary Information

Time-encoded bio-fluorochromic supramolecular co-assembly for rewritable security printing

*Zhao Gao,[a] Shuai Qiu,[a] Fei Yan,[a] Shuyi Zhang,[a] Feng Wang[b] and Wei Tian**

[a]Shaanxi Key Laboratory of Macromolecular Science and Technology, MOE Key Laboratory of Material Physics and Chemistry under Extraordinary Conditions, School of Chemistry and Chemical Engineering, Northwestern Polytechnical University, Xi'an 710072, P. R. China. E-mail: happytw_3000@nwpu.edu.cn
[b]CAS Key Laboratory of Soft Matter Chemistry, iChEM (Collaborative Innovation Center of Chemistry for Energy Materials), Department of Polymer Science and Engineering, University of Science and Technology of China, Hefei, Anhui 230026, P. R. China.

Contents

1. *Materials and methods* S2
2. *Synthetic routes to the targeted monomer* ***1*** S4
3. *Self-assembly behaviors of* ***1*** S6
4. *ATP-driven supramolecular co-assembly* ***1****/ATP* S9
5. *Enzyme mediated transient assembly* S13
6. *Rewritable security printing from* ***1****/ATP/ALP* S15
7. *References* S17
8. *NMR spectra for new compounds* S18

1. Materials and methods

9,10-diiodoanthracene,[S1] compound **4**,[S2] and compound **5** [S3] (see Scheme S1) were synthesized according to the previously reported literatures. Other reagents and solvents used in the experiments were purchased from the commercial sources without further purification.

^{1}H NMR and ^{13}C NMR spectra were obtained from Bruker Avance 400 instruments. Matrix-assisted laser desorption/ionization time-of-flight mass spectrometry mass (MALDI-TOF-MS) measurements were recorded on a Bruker Autoflex Speed spectrometer with DCTB as the matrix. UV–Vis spectra were performed on a Shimadzu UV-2600 spectrometer, equipped with a S-1700 Peltier-type temperature controller. Fluorescence spectra were recorded on a Hitachi F-4600 FL spectrophotometer. Time-resolved fluorescence lifetime experiments were recorded on an Edinburgh FLS980 transient steady-state fluorescence spectrometer. Transmission electron microscope (TEM) images were recorded on a JEM-2100 electron microscope. Scanning electron microscopy (SEM) experiments were performed on a FEI Verios G4 instrument. Dynamic light scattering (DLS) experiments were conducted on a Brookhaven BI-9000AT instrument. The star-shape 3D model was printed from a Formlabs Form 2 3D printer.

Density functional theory (DFT) theoretical calculations: all of the optimized structures were performed on G09 software packages.[S4] All of the elements were described by the B3LYP/6-31G(d) computational method. There are no imagery frequencies for the optimized geometries.

Molecular dynamics (MD) simulations: MD simulations were performed in all-atom representation based on Charmm36 force field and were carried out using the Gromacs-4.6.7 software package.[S5] The system is a relaxed liquid configuration at 298 K in the constant temperature and constant pressure ensemble. The total run time was 20 ns NPT for the equilibrium MD simulations. We used the relaxed system as a starting configuration. The Nosé-Hoover chain thermostat was used to maintain the equilibrium temperature at 298 K and periodic boundary conditions were imposed on all three

dimensions.[S6] The Particle Mesh-Ewald method was used to compute long-range electrostatics interactions.[S7–S8] A cut-off distance of 1 nm was applied to real-space Ewald interactions. The same value was used for van der Waals interactions. The LINCS algorithm [S9] was applied to constrain bond lengths of hydrogen atoms. A leap-frog algorithm [S10] was used with a time step of 2 fs.

*Mathematical fitting of the supramolecular self-assembly process of **1***: the self-assembly of **1** is described by an isodesmic model.[S11] The model is used to describe the self-assembly of **1**, which shows a sigmoidal melting curve as shown in the temperature-dependent UV–Vis experiments. To acquire detailed thermodynamic parameters for the self-assembly process of **1**, the normalized UV–Vis melting curves obtained by plotting the fraction of aggregated species (α_{agg}, λ = 490 nm) against temperature are fitted with the mathematical model.

Inkjet printing experiments: the printing experiments were performed on a commercially available inkjet printer (HP Deskjet 2131) with the customized HP803 black inkjet cartridge. The original black inks from the cartridge were removed, and the cartridge was washed with ethanol, water and dried with N_2 blowing. The security inks of **1** (0.5 mL, 2.0 mM) and ATP (0.5 mL, 2.0 mM) were then loaded separately into the clean black cartridge to perform the printing experiments.

2. Synthetic routes to the targeted monomer 1

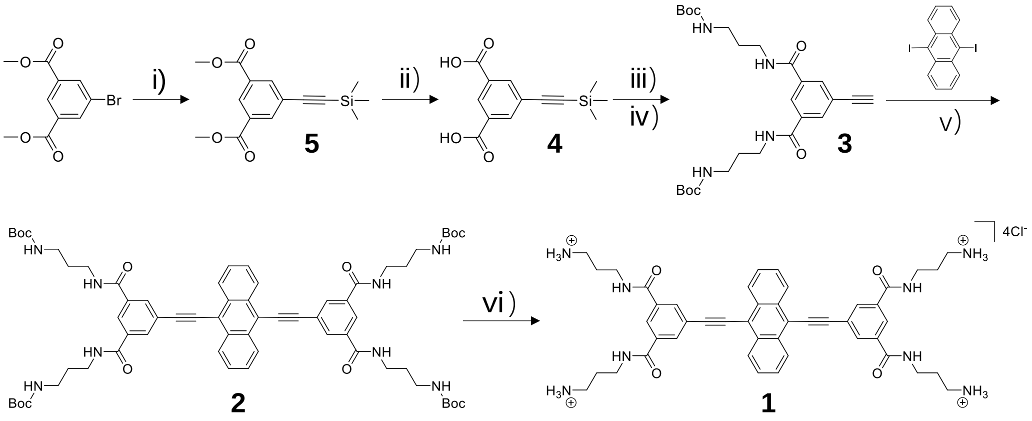

Scheme S1. Synthetic routes to the targeted monomer **1**. i) $Pd(PPh_3)_2Cl_2$, CuI, trimethylsilylacetylene, THF; ii) NaOH, MeOH; iii) EDC, DMAP, CH_2Cl_2; iv) tetrabutylammonium hydroxide, THF; v) $Pd(PPh_3)_2Cl_2$, CuI, TEA; vi) HCl, dioxane, acetone.

*2.1 Synthesis of compound **3***

N-Boc-1,3-propanediamine (696 mg, 4.00 mmol), compound **4** (400 mg, 1.52 mmol)，EDC (1-(3-Dimethylaminopropyl)-3-ethylcarbodiimide hydrochloride) (560 mg, 2.92 mmol) and DMAP (4-dimethylaminopyridine) (720 mg, 5.9 mmol) were mixed in 20 mL of CH_2Cl_2. After stirring at room temperature for 12 hours, the mixture was extracted with H_2O/CH_2Cl_2 for three times. The combined organic extracts were dried over anhydrous Na_2SO_4, and the solvent was removed with a rotary evaporator. The residue was purified by flash column chromatography (petroleum ether/CH_2Cl_2, 5 : 1 *v*/*v* as the eluent) to afford the esterification product (752 mg, 87 %). It was then treated with tetrabutylammonium hydroxide (647 mg, 2.48 mmol) in 20 mL of THF at room temperature for 6 hours. After the deprotection reaction, the solvent was removed in vacuo, and the residue was extracted with H_2O/CH_2Cl_2. The combined organic extracts were dried over anhydrous Na_2SO_4 and evaporated with a rotary evaporator. The residue was purified by flash column chromatography (petroleum ether/CH_2Cl_2, 5 : 1 *v*/*v* as the eluent) to afford compound **3** as a white solid (566 mg, 86 %). ^{1}H NMR (400 MHz, $CDCl_3$) δ (ppm): 8.26 (s, 1H), 8.16 (d, J = 18.9 Hz, 2H), 7.41 (s, 2H), 4.97 (s, 2H), 3.52 (dd, J = 11.9, 6.0 Hz, 4H), 3.29–3.21 (m, 4H), 3.15 (s, 1H), 1.77–1.70 (m,

4H), 1.44 (s, 18H). ^{13}C NMR (101 MHz, $CDCl_3$) δ (ppm): 166.02, 156.90, 136.42, 135.01, 133.62, 123.31, 82.00, 79.62, 78.88, 37.17, 36.48, 29.90, 28.37. MALDI-TOF-MS *m*/*z*: $[M+H]^+$, $C_{26}H_{39}N_4O_6$, calculated 503.2870; found 503.2913.

2.2 Synthesis of compound ***2***

9,10-diiodoanthracene (97.0 mg, 0.23 mmol), compound **3** (258 mg, 0.51 mmol), $Pd(PPh_3)_2Cl_2$ (30mg, 0.04 mmol) and CuI (25.0 mg, 0.13 mmol) were mixed in 20 mL of TEA under nitrogen atmosphere. After stirring at 75 ºC for 12 hours, the reaction mixture was evaporated to remove the solvent, and the residue was extracted with H_2O/CH_2Cl_2 for three times. The combined organic extracts were dried over anhydrous Na_2SO_4, and the solvent was removed with a rotary evaporator. The residue was purified by flash column chromatography (petroleum ether/CH_2Cl_2, 5 : 1 *v*/*v* as the eluent) to afford compound **2** as a yellow solid (153 mg, 57 %). ^1H NMR (400 MHz, DMSO-d_6) δ (ppm): 8.85–8.71 (m, 8H), 8.44 (s, 4H), 8.40 (s, 2H), 7.88 (s, 4H), 6.87 (s, 4H), 3.02 (d, J = 6.1 Hz, 8H), 1.75–1.63 (m, 8H), 1.38 (s, 36H). ^{13}C NMR (101 MHz, $CDCl_3$) δ (ppm): 166.21, 165.65, 156.83, 136.35, 134.93, 133.54, 133.23, 125.24, 123.63, 123.14, 123.02, 81.93, 79.55, 78.92, 37.21, 36.60, 29.73, 28.33. MALDI-TOF-MS *m*/*z*: $[M+H]^+$, $C_{66}H_{83}N_8O_{12}$, calculated 1179.6130; found 1179.6535.

2.3 Synthesis of monomer ***1***

A concentrated HCl solution was added into a dioxane (10 mL) solution of compound **2** (153 mg, 0.13 mmol) with vigorous stirring at 0 ºC under N_2 atmosphere. The mixture was allowed to warm slowly to room temperature and stirred for 24 h. The yellow precipitation was then washed with dioxane for multiple times, and dry to obtain monomer **1** as a yellow solid (105 mg, 87%). ^1H NMR (400 MHz, DMSO-d_6) δ (ppm): 9.11 (t, J = 5.5 Hz, 4H), 8.80 (dd, J = 6.6, 3.3 Hz, 4H), 8.55 (s, 2H), 8.53 (s, 4H), 7.92 (s, 8H), 7.88 (dd, J = 6.7, 3.2 Hz, 4H), 3.47–3.38 (m, 8H), 2.91 (d, J = 6.4 Hz, 8H), 1.95–1.83 (m, 8H). ^{13}C NMR (101 MHz, DMSO-d_6) δ (ppm): 168.34, 135.65, 133.97, 132.48, 129.13, 127.90, 127.48, 124.76, 118.60, 102.35, 89.00, 38.31, 38.08, 28.19. MALDI-TOF-MS *m*/*z*: $[M–4Cl]^{4+}$, $C_{46}H_{54}N_8O_4$, calculated 195.6062; found 195.6058.

3. Self-assembly behaviors of 1

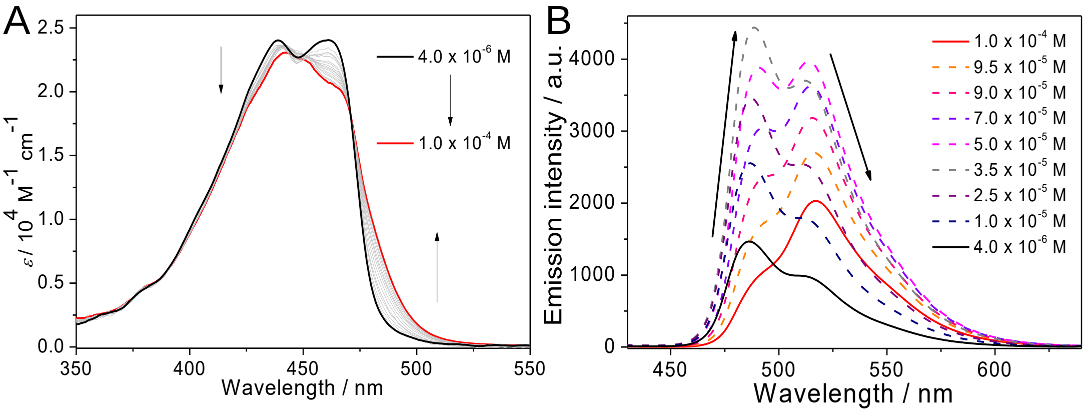

Figure S1. Self-assembly behaviors of **1**. Concentration-dependent (A) UV–Vis absorption and (B) fluorescence spectra of **1** in aqueous solution from 4.0 × 10^{-6} M to 1.0 × 10^{-4} M. λ_{ex} = 320 nm. Upon gradually increasing the concentration of **1**, the molar extinction coefficient (ε) at around 460 nm gradually decreases, while a red-shifted shoulder band locating between 470 and 530 nm appears. Two distinctive isosbestic points at 471 and 375 nm suggest the transition from molecularly dissolved state to self-assembled state.

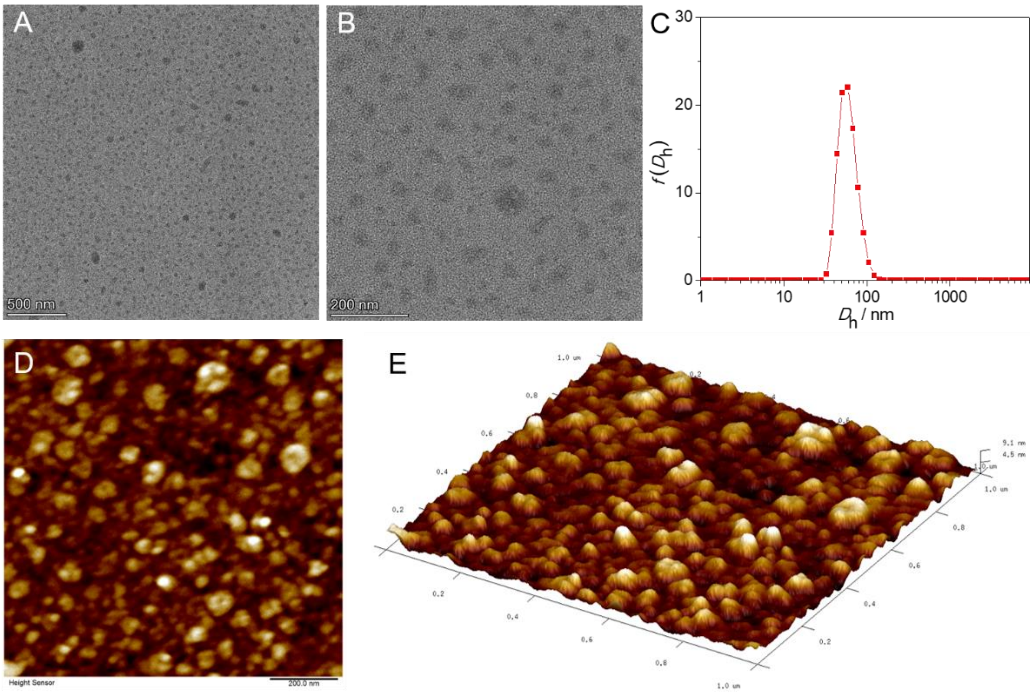

Figure S2. Morphologies characterization for **1**. (A)–(B) TEM images by drop-casting of **1** (2.0 × 10^{-4} M) on the copper grids; (C) DLS measurement of **1** in aqueous solution (1.0 × 10^{-4} M, 298 K); (D)–(E) AFM images of **1** on the silicon wafer. DLS, TEM and AFM measurements reveal that **1** can self-assemble into nano-aggregates (~60 nm DLS hydrodynamic diameter) in aqueous solution.

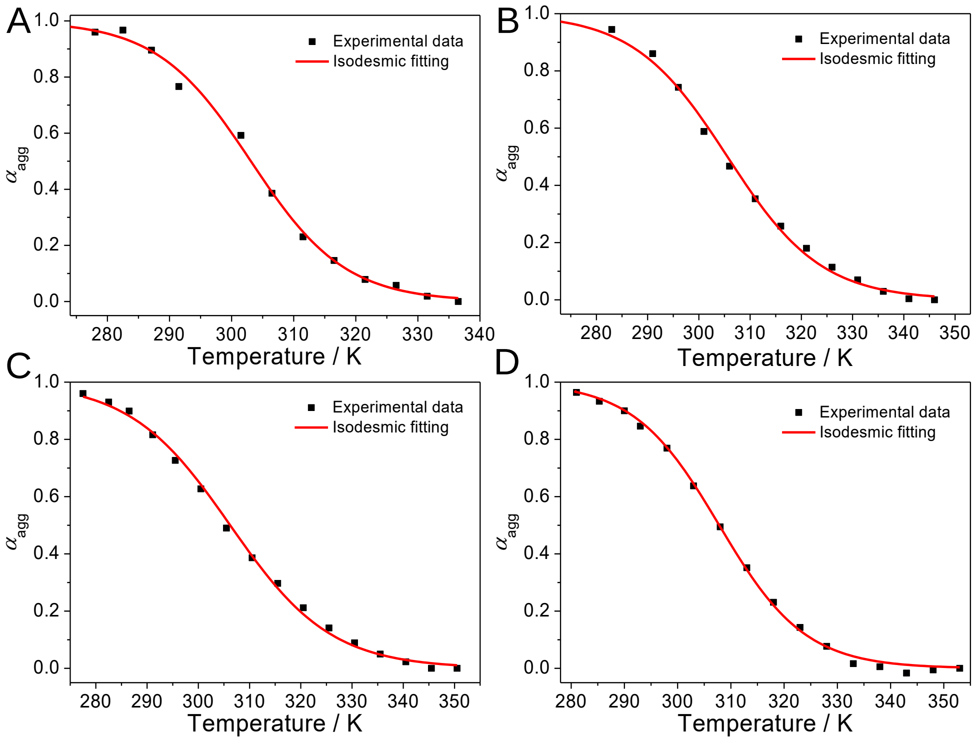

Figure S3. Temperature-dependent UV–Vis absorption spectra of **1** in aqueous solution. (A) 0.8×10^{-4} M, (B) 1.0×10^{-4} M, (C) 1.2×10^{-4} M, (D) 1.4×10^{-4} M. The normalized melting curves at $\lambda = 490$ nm display sigmoidal curves, which are characteristic for the isodesmic assembling mechanism. Non-linear fitting of the normalized curve affords the corresponding thermodynamic parameters, as shown in Table S1.

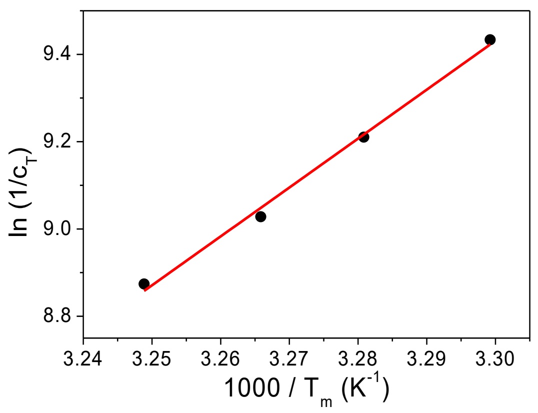

Figure S4. Van’t Hoff plots of **1**. The red line denotes the respective linear fitting curve. According to the Van’t Hoff plot, ΔG of the self-assembly process of **1** is calculated to be –24.9 kJ mol^{-1} at 298 K.

Table S1. Thermodynamic parameters of the self-assembly process of 1 in aqueous solution. The data are obtained by fitting the temperature-dependent UV–Vis absorption data.

Concentration [M]	T_m[a)] [K]	ΔH[b)] [kJ mol^{-1}]
0.8×10^{-4}	303.1	–101.9
1.0×10^{-4}	304.8	–100.9
1.2×10^{-4}	306.2	–87.5
1.4×10^{-4}	307.8	–107.7

[a)]Melting temperature at which α_{agg} is 0.5; [b)]Enthalpy release upon aggregation.

4. ATP-driven supramolecular co-assembly 1/ATP

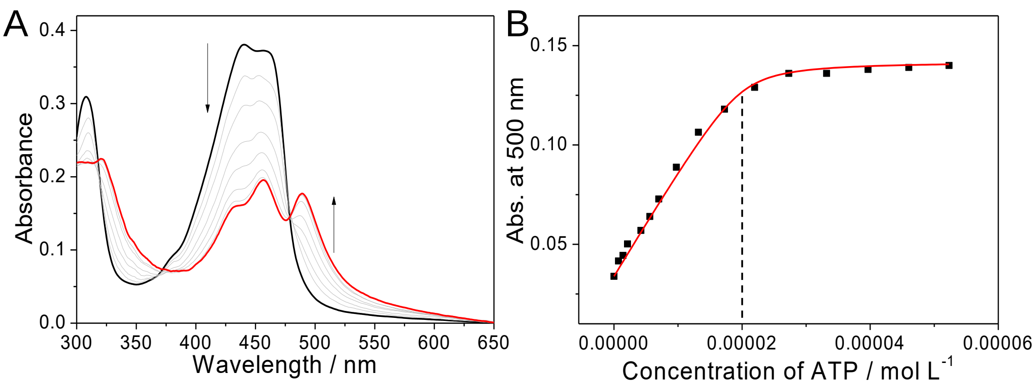

Figure S5. (A) UV–Vis spectra variations upon gradual addition of ATP ([ATP] = 2.0 × 10^{-3} M) into **1** ([**1**] = 2.0 × 10^{-5} M). (B) the absorbance intensity changes at λ = 500 nm. The red line indicates the nonlinear curve fitting according to the 1:1 binding model. Molar ratio plot is acquired by monitoring the absorbance intensity at 500 nm, providing the clear evidence for 1 : 1 binding stoichiometry between **1** and ATP.

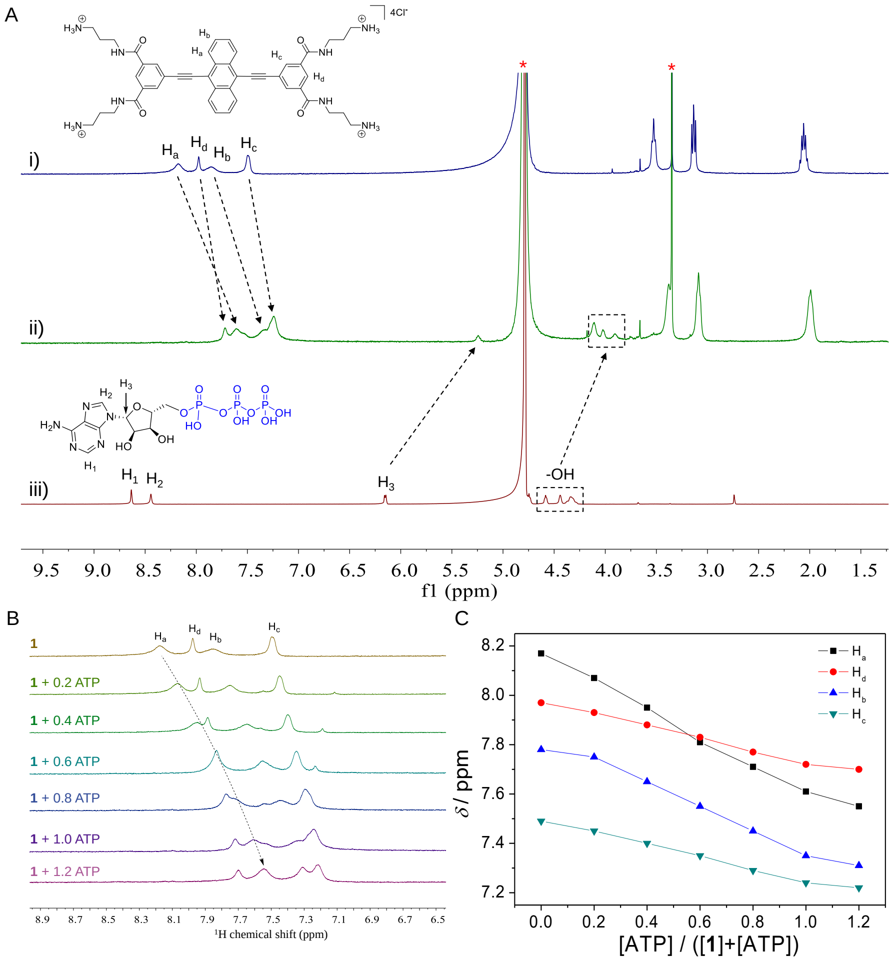

Figure S6. ^{1}H NMR spectra characterization for ATP-driven supramolecular co-assembly. (A) Partial ^{1}H NMR spectra (300 MHz, 298 K, 2.00 mM) of i) **1**, ii) **1**/ATP

([**1**] : [ATP] = 1 : 1), and iii) ATP in D_2O. (B) Partial ^{1}H NMR spectra (300 MHz, 298 K, 2.00 mM): **1** in D_2O at the presence of 0–1.2 equiv of ATP. The arrow shows the gradual upfield shifting of the H_a proton. (C) $H_{a\text{–}d}$ resonances change *versus* the molar ratio of **1** and ATP.

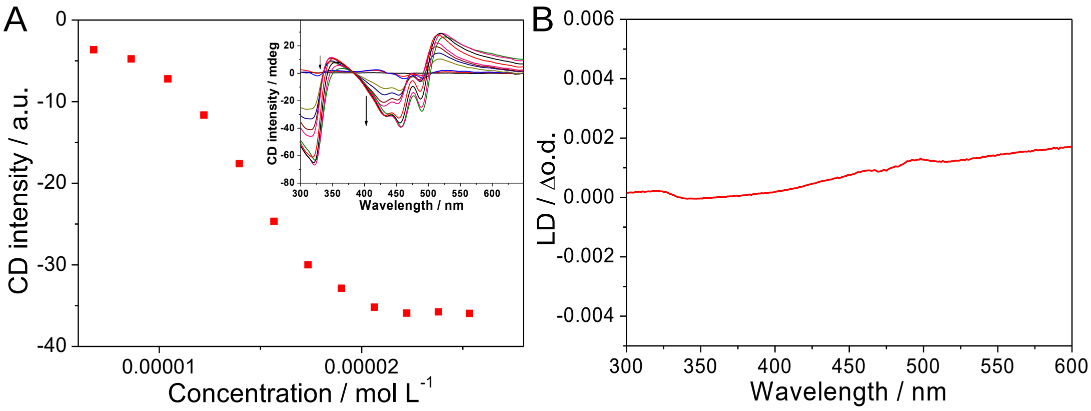

Figure S7. Supramolecular chiral characterization for **1**/ATP. (A) CD intensity changes at λ = 459 nm upon gradual addition of ATP ([ATP] = 2.0 × 10^{-3} M) into **1** ([**1**] = 2.0 × 10^{-5} M). Inset of (A): CD spectra variations upon gradual addition of ATP into **1**. (B) LD signal of **1**/ATP ([**1**] 2.0 × 10^{-5} M, [ATP] = 2.0 × 10^{-5} M) in HEPES buffer. Upon progressively adding ATP into **1**, the Cotton effect gradually appears, which indicates that ATP induces a preferred helical handedness to the resulting co-assembly of **1**/ATP. Negligible LD signals are observed for the co-assembly of **1**/ATP. Therefore, the measured CD signals are real to reflect the supramolecular chirality, without the interference of LD.

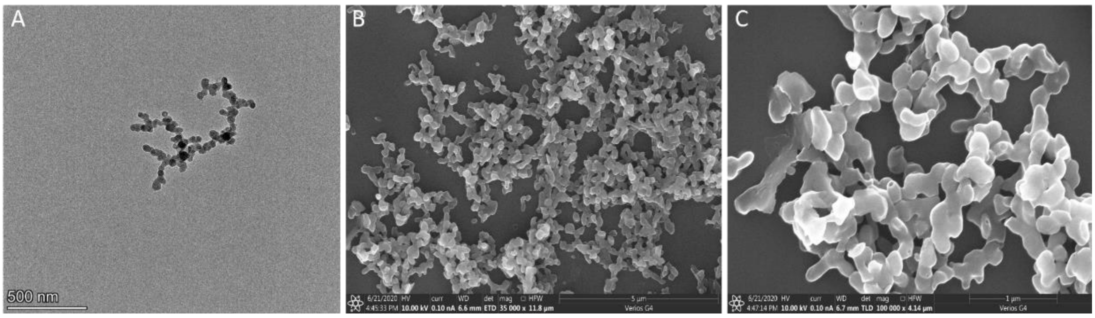

Figure S8. Morphologies characterization for **1**/ATP. (A) TEM and (B)–(C) SEM micrographs of **1**/ATP ([**1**] : [ATP] = 1 : 1).

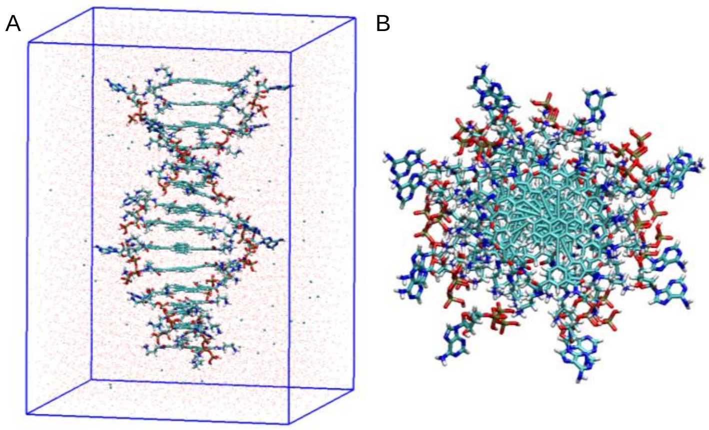

Figure S9. MD simulations for **1**/ATP. (A) Side view and (B) top view of the structure of the co-assembly **1**/ATP obtained from MD simulations at the initial snapshots. The red dots in (A) represent the water molecules.

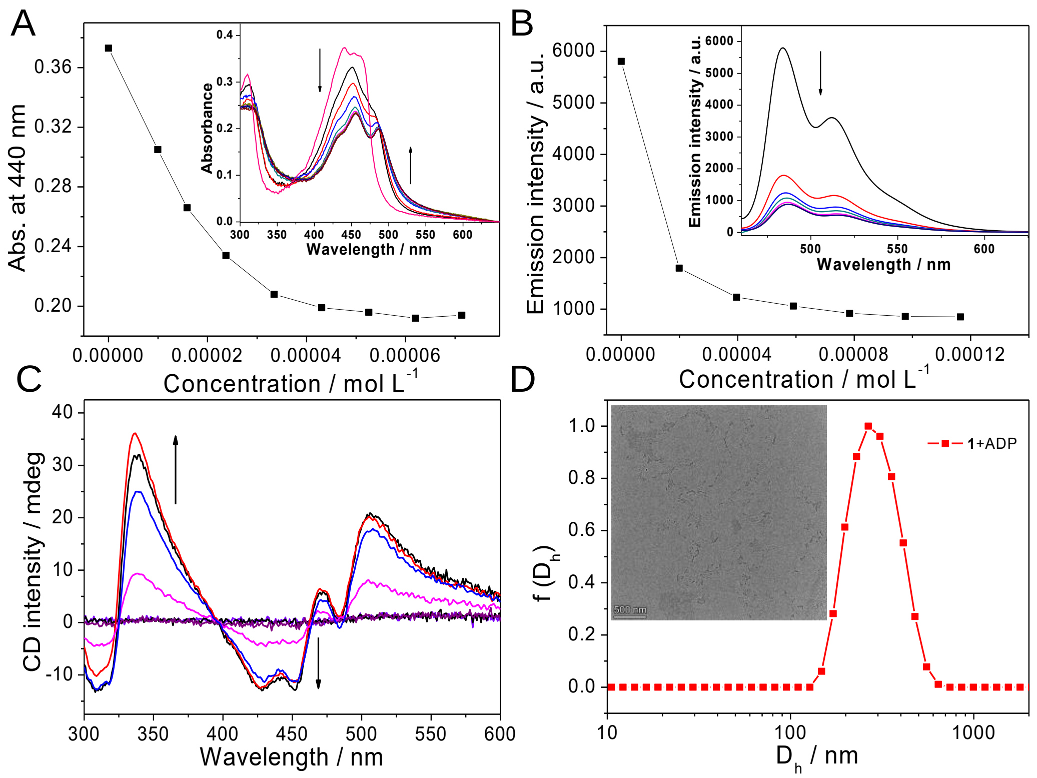

Figure S10. Co-assembly behaviors of **1**/ADP. (A) UV–Vis, (B) fluorescence, (C) CD, and (D) DLS spectra variations upon gradual addition of ADP ([ADP] = 2.0×10^{-3} M in HEPES buffer) into **1** (2.0×10^{-5} M in HEPES buffer). Specifically, around two equivalents of ADP are required to reduce the absorbance and emission intensities. Moreover, ADP can also induce CD signals with an isodichroic point at 397 nm, but less intense to that of ATP. Only small aggregations are formed for the resulting co-assembly **1**/ADP ([**1**] : [ADP] = 1 : 2), as evidenced by the TEM and DLS measurements.

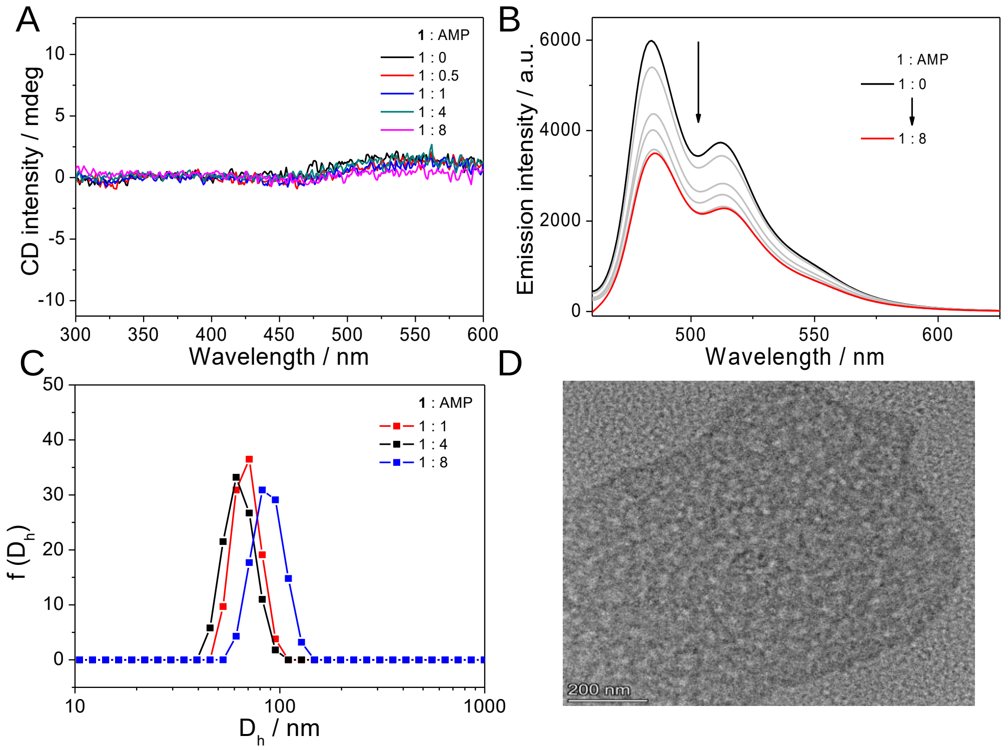

Figure S11. Co-assembly behaviors of **1**/AMP. (A) CD, (B) fluorescence, and (C) DLS spectra variations upon gradual addition of AMP into **1** (2.0×10^{-5} M in HEPES buffer). (D) TEM image of **1**/AMP ([**1**] : [ATP] = 1 : 8). Even though an excess of AMP is added into **1**, no apparent co-assembly can be observed.

5. Enzyme mediated transient assembly

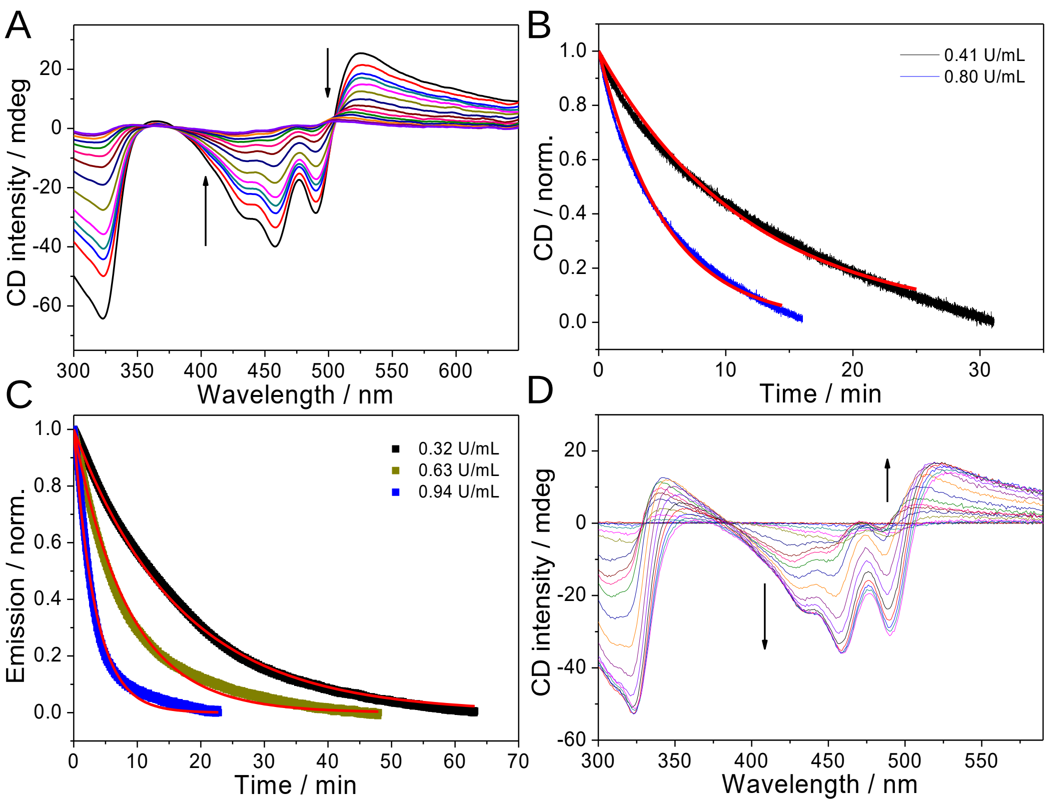

Figure S12. ALP mediated transient co-assembly of **1**/ATP ([**1**] = 2.0 × 10^{-5} M, [ATP] = 2.0 × 10^{-5} M, aqueous HEPES buffer, pH = 7.0). (A) CD spectra variations of **1**/ATP *versus* hydrolysed time in the presence of 0.32 U mL^{-1} of ALP. (B) Time-dependent CD intensity monitored at 458 nm with varying ALP concentration (0.41 and 0.80 U mL^{-1}). ([**1**] = 2.0 × 10^{-5} M, [ATP] = 2.0 × 10^{-5} M, aqueous HEPES buffer, pH = 7.0). The solid lines correspond to their first-order kinetic fits. CD signals are normalized between zero and one for the ease of fitting into kinetic model. Time-dependent CD spectra show a weakening trend during the enzymatic process of **1**/ATP by ALP, indicating the gradual disassembly of the chiral aggregates. When the amount of ALP varies from 0.41 U mL^{-1} to 0.80 U mL^{-1}, the corresponding rate constants of disassembly are calculated to be 0.08 min^{-1} and 0.19 min^{-1}, respectively. (C) Time-dependent emission intensity monitored at 484 nm showing the disassembly process of **1**/ATP, with varying ALP concentration. ([**1**] = 2.0 × 10^{-5} M, [ATP] = 2.0 × 10^{-5} M, aqueous HEPES buffer, pH = 7.0). When the amount of ALP increases from 0.32 U mL^{-1} *via* 0.63 U mL^{-1} to 0.94 U mL^{-1}, the recovery half-life time (defined as the time required to reach 50% of the fluorescence intensity) shortens from 14.5 min *via* 7.3 min to 3.6 min. The corresponding rate constants of disassembly are calculated to be 0.06 min^{-1}, 0.12 min^{-1}, and 0.29 min^{-1}. (D) CD spectra variations upon re-addition of ATP (2.0 × 10^{-5} M) into the mixture solution of **1**/ALP. The Cotton effects totally restore to the initial state as a function of time, which indicates the chiral co-assembly **1**/ATP is re-formed.

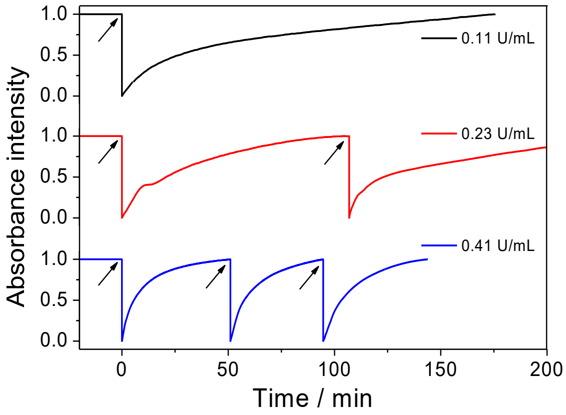

Figure S13. Time-dependent absorbance intensity at 442 nm on several repetitive additions of ATP to **1** in the presence of 0.11 U mL^{-1}, 0.23 U mL^{-1}, and 0.41 U mL^{-1} of ALP, respectively. Arrows represent the (re)fueling points with the addition of ATP. ([**1**] = 2.0×10^{-5} M, [ATP] = 2.0×10^{-5} M, aqueous HEPES buffer, pH = 7.0). Upon increasing the amount of prestored ALP in **1**/ATP, the shortened half-life times of the transient cycles are observed from the time-dependent absorption spectra.

6. Rewritable security printing from 1/ATP/ALP

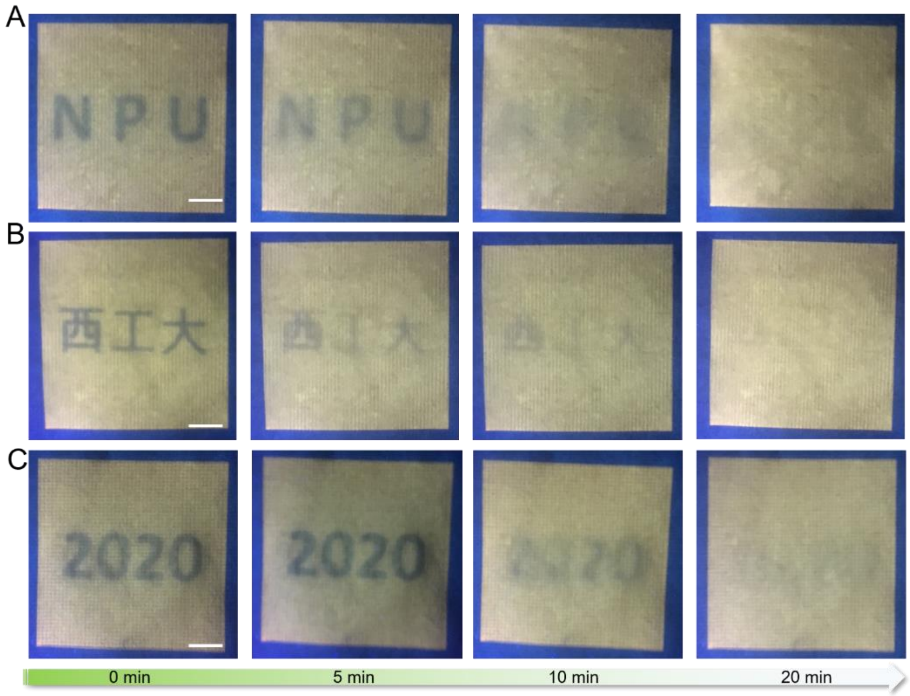

Figure S14. Photographs of the printed patterns derived from **1**, ATP and ALP. The yellow emission background is printed with the aqueous solution of **1** as inks. (A) The "NPU" pattern that printed with the inks of ATP is gradually disappeared after spraying with ALP inks. (B) The reprinted pattern of Chinese characters with ATP inks at the same location of (A), is gradually disappeared after spraying with ALP inks. (C) The reprinted pattern of "2020" with ATP inks at the same location of (B), is gradually disappeared after spraying with ALP inks. [ALP] = 0.80 U mL^{-1} Scale bars: 5 mm.

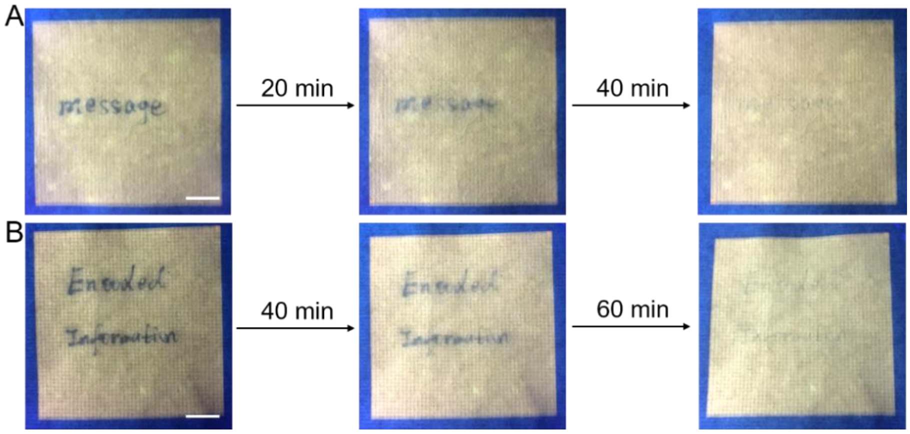

Figure S15. Regulation of the erasing time of the rewritable information. The word of (A) "message" and (B) "Encoded Information" that handwritten with a fountain pen loaded with the ATP inks are gradually disappeared after spraying with different concentration of ALP inks (0.6 U mL^{-1} for (A), and 0.4 U mL^{-1} for (B)), respectively. Scale bars: 5 mm. The erasing time of the rewritable information can be regulated

according to the concentration of ALP inks, endowing with security in the time dimension.

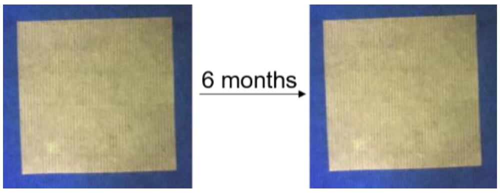

Figure S16. The stability study for **1**/ATP. Photographs of the printed yellow emission paper with the aqueous solution of **1** as inks on day 1 and after 6 months. The rewritable paper features enough stability under the atmosphere condition, as evidenced by the unchanging of the emission color for at least half a year.

7. References

[S1] M. Lebtow, I. Helmers, V. Stepanenko, R. Q. Albuquerque, T. B. Marder and G. Fernández, *Chem. Eur. J.*, 2017, **23**, 6198.

[S2] D. Zhao, D. Yuan, R. Krishna, J. M. van Batenb and H.-C. Zhou, *Chem. Commun.*, 2010, **46**, 7352.

[S3] K. Shao, J. Pei, J.-X. Wang, Y. Yang, Y. Cui, W. Zhou, T. Yildirim, B. Li, B. Chen and G. Qian, *Chem. Commun.*, 2019, **55**, 11402.

[S4] Gaussian 09, Revision A.02, M. J. Frisch, G. W. Trucks, H. B. Schlegel, G. E. Scuseria, M. A. Robb, J. R. Cheeseman, G. Scalmani, V. Barone, B. Mennucci, G. A. Petersson, H. Nakatsuji, M. Caricato, X. Li, H. P. Hratchian, A. F. Izmaylov, J. Bloino, G. Zheng, J. L. Sonnenberg, M. Hada, M. Ehara, K. Toyota, R. Fukuda, J. Hasegawa, M. Ishida, T. Nakajima, Y. Honda, O. Kitao, H. Nakai, T. Vreven, J. A. Montgomery, Jr., J. E. Peralta, F. Ogliaro, M. Bearpark, J. J. Heyd, E. Brothers, K. N. Kudin, V. N. Staroverov, R. Kobayashi, J. Normand, K. Raghavachari, A. Rendell, J. C. Burant, S. S. Iyengar, J. Tomasi, M. Cossi, N. Rega, J. M. Millam, M. Klene, J. E. Knox, J. B. Cross, V. Bakken, C. Adamo, J. Jaramillo, R. Gomperts, R. E. Stratmann, O. Yazyev, A. J. Austin, R. Cammi, C. Pomelli, J. W. Ochterski, R. L. Martin, K. Morokuma, V. G. Zakrzewski, G. A. Voth, P. Salvador, J. J. Dannenberg, S. Dapprich, A. D. Daniels,O. Farkas, J. B. Foresman, J. V. Ortiz, J. Cioslowski, and D. J. Fox, *Gaussian, Inc., Wallingford CT*, 2009.

[S5] B. Hess, C. Kutzner, D. van der Spoel amd E. Lindahl, *J. Chem. Theory Comput.*, 2008, **4**, 435.

[S6] H. J. Berendsen, J. P. M. Postma, W. F. van Gunsteren, A. DiNola and J. Haak, *J. Chem. Phys.*, 1984, **81**, 3684.

[S7] U. Essmann, L. Perera, M. L. Berkowitz, T. Darden, H. Lee and L. G. Pedersen, *J. Chem. Phys.*, 1995, **103**, 8577.

[S8] L. G. Astrakas, C. Gousias and M. J. Tzaphlidou, *Appl. Phys.*, 2012, **111**, 074702.

[S9] B. Hess, H. Bekker, H. J. Berendsen and J. G. Fraaije, *J. Comput. Chem.*, 1997, **18**, 1463.

[S10] W. F. Van Gunsteren and H. Berendsen, *Mol. Simul.*, 1988, **1**, 173.

[S11] M. M. J. Smulders, M. K. L. Nieuwenhuizen, T. F. A. de Greef, P. van der Schoot, A. P. H. J. Schenning and E. W. Meijer, *Chem. Eur. J.,* 2010, **16**, 362.

8. NMR spectra for new compounds

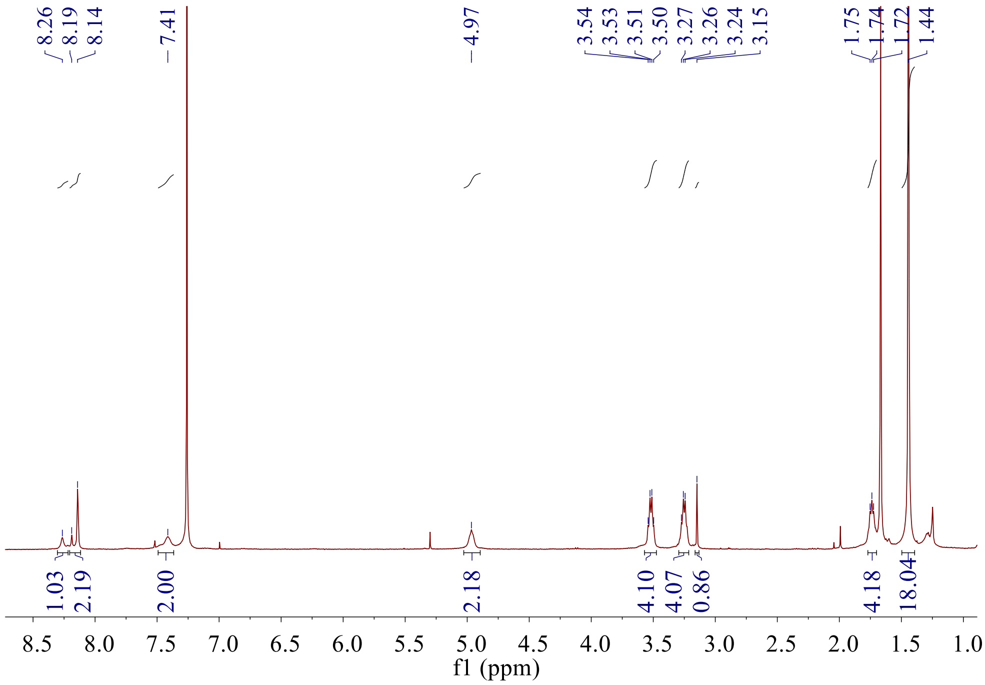

Figure S17. ^{1}H NMR spectrum (400 MHz, $CDCl_3$, 298 K) of compound **3**.

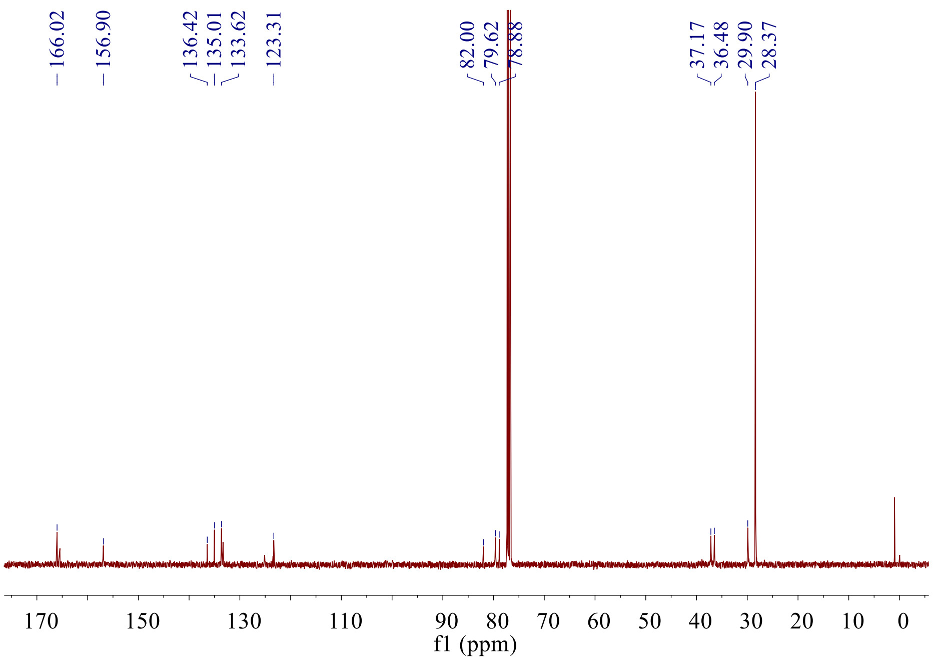

Figure S18. ^{13}C NMR spectrum (101 MHz, $CDCl_3$, 298 K) of compound **3**.

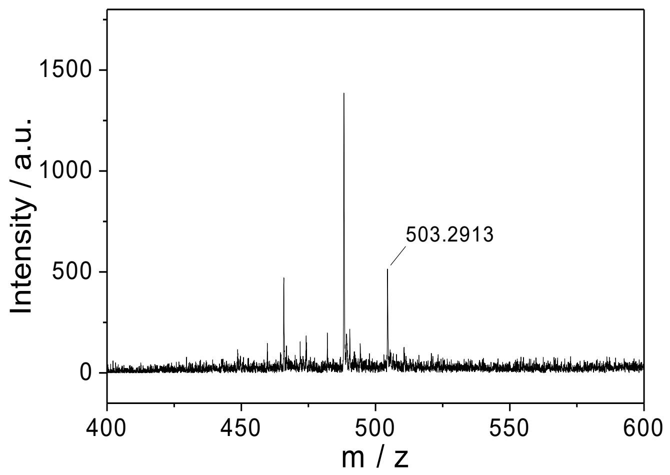

Figure S19. MALDI-TOF-MS spectrum of compound **3**.

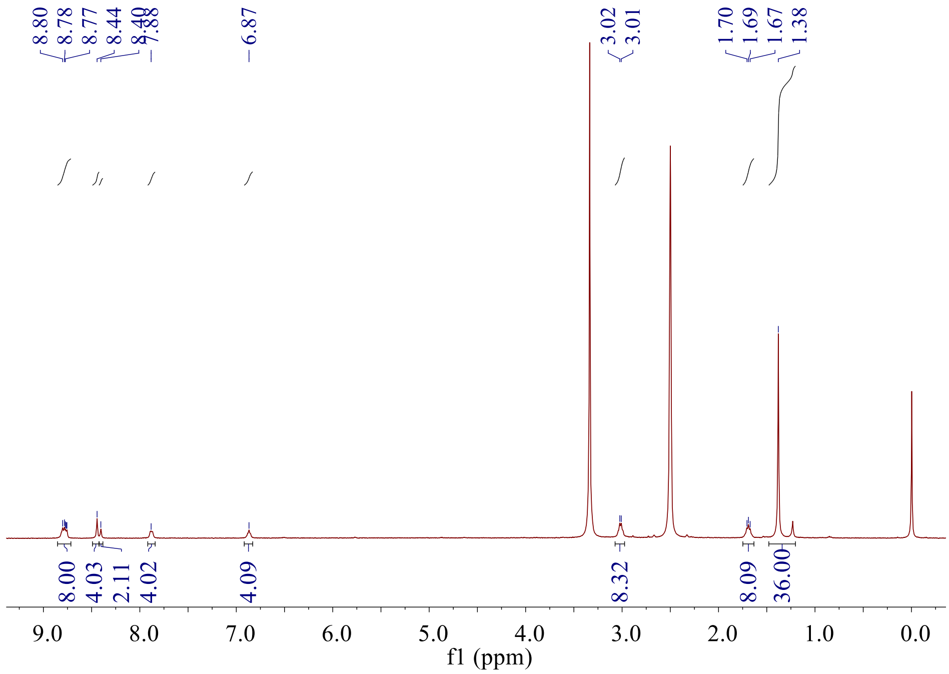

Figure S20. ^{1}H NMR spectrum (400 MHz, DMSO-d_6, 298 K) of compound **2**.

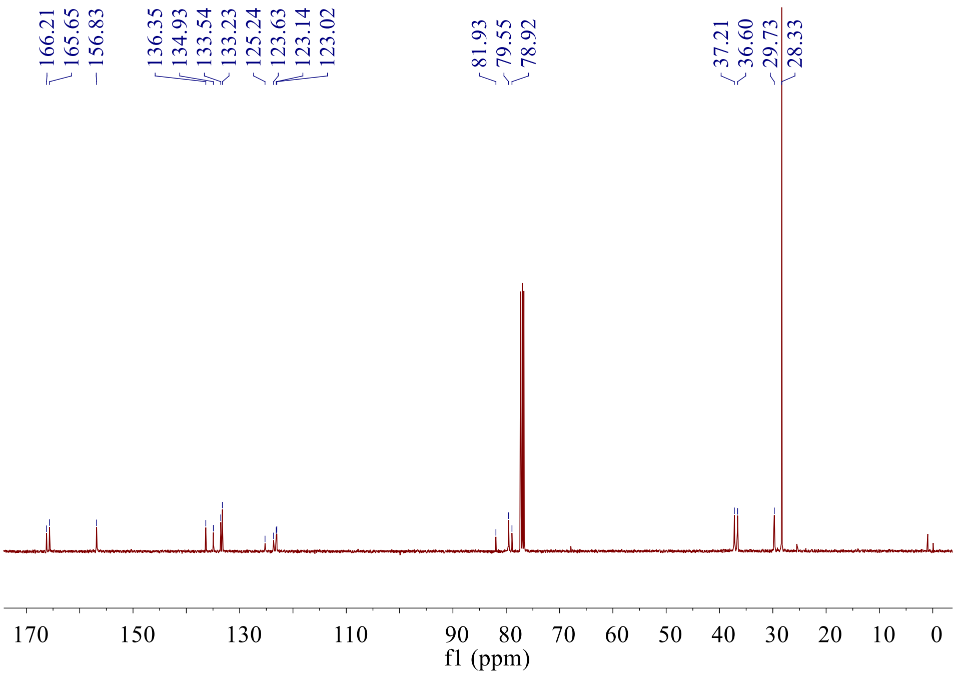

Figure S21. ^{13}C NMR spectrum (101 MHz, $CDCl_3$, 298 K) of compound **2**.

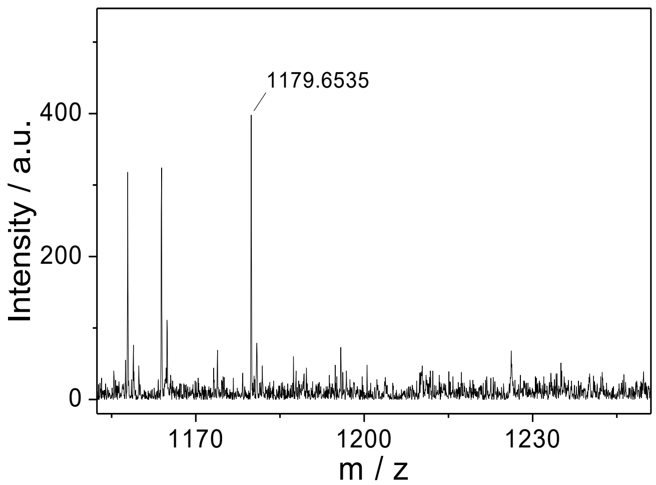

Figure S22. MALDI-TOF-MS spectrum of compound **2**.

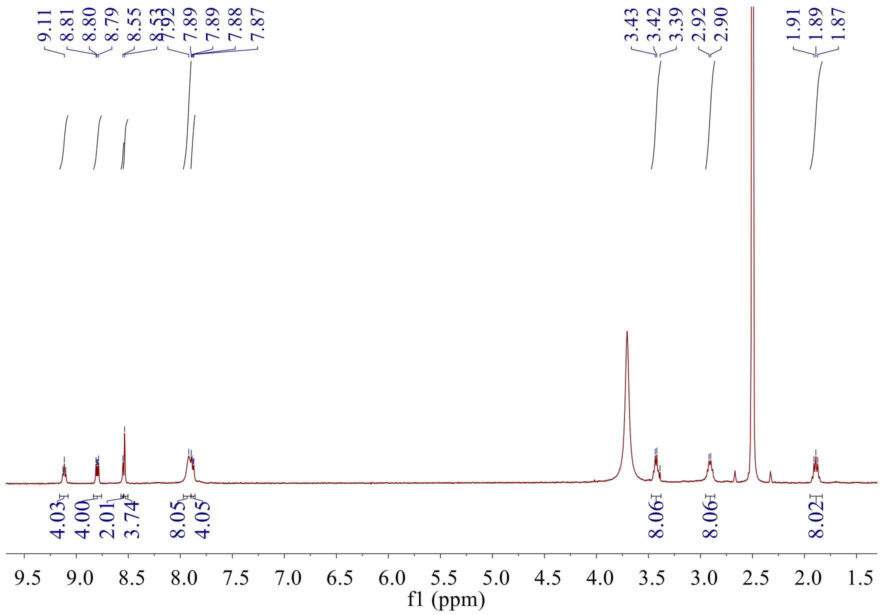

Figure S23. ^{1}H NMR spectrum (400 MHz, DMSO-d_6, 298 K) of monomer **1**.

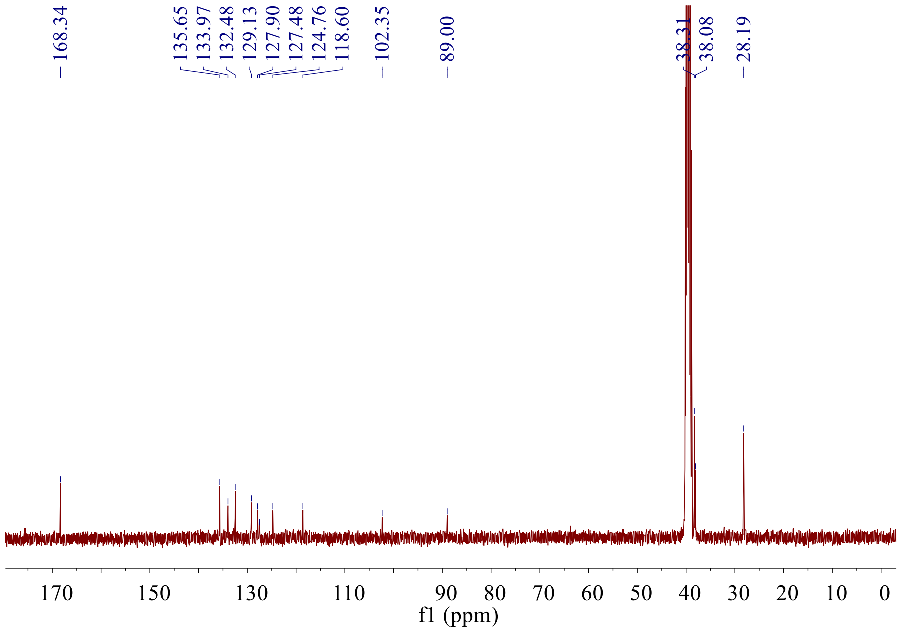

Figure S24. ^{13}C NMR spectrum (101 MHz, DMSO-d_6, 298 K) of monomer **1**.

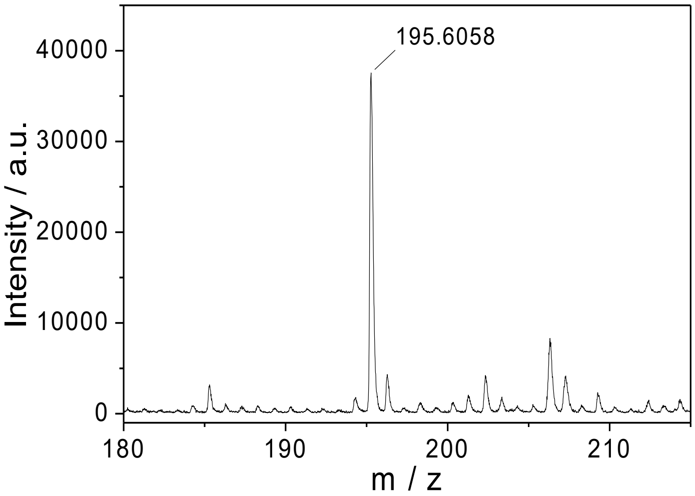

Figure S25. MALDI-TOF-MS spectrum of monomer **1**.
